# The Potential of *Jatropha variegata* Fruits as a Natural Contraceptive: Antifertility Activity and Phytochemical Analysis

**DOI:** 10.1155/2022/1365526

**Published:** 2022-02-21

**Authors:** Wahibah Taher Alhaj, Bushra Abdulkarim Moharram, Tareq Al-Maqtari, Hassan M. Al-Mahbashi, Amin A. Al-Doaiss

**Affiliations:** ^1^Department of Pharmacognosy, Faculty of Pharmacy, Sana'a University, Sana'a, Yemen; ^2^Department of Pharmacology, Faculty of Pharmacy, Sana'a University, Sana'a, Yemen; ^3^Department of Forensic Medicine and Clinical Toxicology, Faculty of Medicine, Sana'a University, Sana'a, Yemen; ^4^Anatomy and Histology Department, Faculty of Medicine, Sana'a University, Sana'a, Yemen

## Abstract

**Background:**

*Jatropha variegata* (family, Euphorbiaceae) is native to Yemen, where it is commonly known as the Ebki shrub. The fruits of the plant are traditionally ingested by local women as a natural method of contraception. This study was undertaken to investigate the phytochemical content of the methanol extract of *J. variegata* fruits and to evaluate its antifertility potential.

**Methods:**

Isolation of the chemical constituents was performed by chromatographic techniques, and the chemical structures of these compounds were identified by spectroscopy. The antifertility activity of the methanol extract was assessed in two experimental rat models to explore both the anti-implantation and the estrogenic/antiestrogenic activities in females. In these models, the number of successful implants, the size of litter, and body/ovary weights were all recorded. The development of ovarian follicles was also monitored *via* histological staining.

**Results:**

Phytochemical work on the fruit extract of *J. variegata* led to the isolation of two oils (JF1 and JF2) and methyl elaidate. GC-MS analysis of the JF1 oil revealed that the major chemical constituents were fatty acid esters (43.77%), hydrocarbon alkanes (20.65%), and terpenoids (4.65%), while terpenoids (28.8%), fatty acids and their esters, (29.47%), and phytosterol (10.49%) were the major components found in the JF2 oil. The methanol extract of *J. variegata* fruit exhibited 50% and 93% abortifacient activity at 150 and 300 mg/kg doses, respectively. The extract also showed significant estrogenic activity as evidenced by the increase in rat ovary weight at a dose of 300 mg/kg compared to the control group. Histological analyses further confirmed this estrogenic activity.

**Conclusions:**

*J. variegata* fruits possess an antifertility activity that appeared to result from its antiembryo implantation potential and from its estrogenic activity. The bioactive constituents involved in these activities may need to be further explored and exploited in the pursuit of newer contraceptives.

## 1. Introduction

*Jatropha variegata* (Forsk.) Vahl (family; Euphorbiaceae) is a species native to Yemen [1]. It is traditionally used in Yemeni folk medicine, similar to other *Jatropha* species, in the management of wounds [2] and as a contraceptive [3]. *Jatropha* species have been shown to possess cytotoxicity, antitumor, antimicrobial, antiprotozoal, anticoagulant, immunomodulating, antioxidant, anti-inflammatory, molluscicidal, AchE inhibitory, protoscolicidal, insecticidal, and wound healing activities [3–6]. The bioactive components in *Jatropha* species include alkaloids, cyclic peptides, terpenes (monoterpenes, sesquiterpenes, diterpenes, and triterpenes), flavonoids, lignans, coumarins, coumarino-lignoids, a noncyanogenic glucoside, phloroglucinols, ester ferulates, phenolics, deoxypreussomerins, and fatty acids [4, 5].

Population explosion is a great concern worldwide and adversely affects the social, economic, and technological development of individuals and societies, particularly in poor developing countries. In Yemen, the fertility rate, as in many Arab countries, is among the highest in the world. The increasing population growth in Yemen (around 3% each year) had a big toll on the country's economic growth and exacerbated poverty [7, 8]. Thus, family planning programs have been adopted by the successive Yemeni government and various contraceptive methods have been promoted [9].

Although various synthetic contraceptive agents have evidently played a great role in reducing the fertility rate, each one carries along with its share of undesirable side effects [10]. Hence, there is an urgent need to find an alternative to the commonly used synthetic contraceptives and to identify newer effective contraceptive agents with superior safety profiles and hopefully low cost. Drugs of plant origin may afford much of these properties [11–13]. Hence, this study was carried out to scientifically validate the antifertility potential of *Jatropha variegata* fruits in female albino rats and to determine its phytochemical constituents. To the best of our knowledge, this is the first report on the antifertility potential of *J. variegata*, worldwide.

## 2. Materials and Methods

### 2.1. Chemicals, Reagents, and Drugs

The following chemicals and standards were used: methanol (MeOH) (UNI-CHEM, Beograd); ethyl acetate (EtOAc) (HiMedia, India); formaldehyde (WINLAB, UK); estradiol valerate (Progyluton®, Germany); xylazine (XYL-M2®) (Arendonk, Belgium); ketamine 10% (Alfasan Woerden, Holland); and Tween 80 (UNI-CHEM, Beograd).

### 2.2. Plant Materials

Fruits of *Jatropha varigata* Vahl. were collected in August and September of 2018 from Al-Aboos village, Haifan district (1,157 meter altitude), Taiz city, Yemen. The plant was identified by Dr. Abdul Wali Alkulaidi (Public Authority for Research and Agricultural Extension, Dhamar, Yemen). The voucher specimen of the plant was prepared and deposited at the Department of Pharmacognosy, Faculty of Pharmacy, Sana'a University, with a voucher number of JV2 2018.

### 2.3. Experimental Animals

Mature female Albino rats, weighing 150–250 g, were obtained from the animal house of the Faculty of Science, Sana'a University. All rats were handled according to the guidelines for care and use of laboratory animals [14]. The animals were housed in polypropylene cages under controlled temperature (23 ± 2°C) and light (light-dark cycle of 12 h) with food and water ad libitum. The rats were acclimatized to the laboratory at least 10 days before experimentation. The care and handling of animals were in accordance with internationally accepted Ethical Guidelines for Laboratory Animals, National Institutes of Health (NIH) Publication No. 86–23, Revised 1985, and the ARRIVE guidelines.

### 2.4. Preparation of the Methanol Extract

The fruits (295 g) of the plant were cleaned, air-dried in the shade, and then grounded. The powdered material was extracted with 95% methanol using a Soxhlet apparatus (at a temperature not exceeding 45°C). The extract was then filtered, and the filtrate was dried via a rotary evaporator (Buchi Rotavapour R-215; Switzerland) in a water bath (Buchi water bath B-491; Switzerland) at a temperature not exceeding 45°C. The extract yield percentage (7.67%) was calculated based on initial dry weights. The final extract was stored in an airtight container at room temperature.

### 2.5. Phytochemical Study

#### 2.5.1. General Experimental Procedure

UV-lamp (Vilber Lourmat, France) was used to measure absorbance at both 365 nm and at 254 nm. Melting points were determined using electrothermal melting point apparatus—model 9100—obtained from Hitech, Mount Holly, USA. ^1^H NMR and ^13^C NMR spectra (1D and 2D) of compounds were carried out using a Bruker® Avance III HD (^1^H at 400 and ^13^C at 100 MHz) spectrometer (Germany) equipped with a 5 mm broad-band multinuclear (PABBO) probe. In these NMR experiments, dimethyl sulfoxide (DMSO) was the solvent and tetramethylsilane (TMS) served as an internal standard. Column chromatography was carried out on 100–200 mesh silica gel (HiMedia, India). TLC was performed on 20 cm × 20 cm silica gel 60 GF254 plates (Merck, Germany), and 20 cm × 20 cm kieselgel 100 F254 plates (Merck) were used for preparative TLC (PTLC). All solvents were of analytical grade.

#### 2.5.2. Extraction and Isolation

The dried methanol extract (146.89 g) was redissolved in methanol and fractionated using hexane and a separating funnel. The hexane extract was then dried under vacuum and yielded 48.31 g of extract. Ten grams of the hexane extract was then fractionated via silica gel column chromatography (61.5 cm × 1.9 cm, 55.92 g) and was eluted with a solvent gradient of hexane/EtOAc (10 : 0 to 0 : 10), EtOAc/MeOH (10 : 0 to 0 : 10). The eluted fractions were collected and then tested via TLC. All fractions with similar compositions were pooled together, yielding 13 different fractions (HJv1–13). Overnight, fraction **HJv1** (410.0 mg) eluted with 100% hexane and fraction **HJv2** (349 mg) eluted with hexane/EtOAc (9 : 1) both produced white amorphous powder and an oil layer. The powders of both HJv1 and HJv2 were carefully separated from the oil layers to yield **JF1** (106 mg) and **JF2** (21 mg) oils, respectively. The JF1 and JF2 oils were analyzed by GC-MS to determine their chemical composition. However, the powders obtained from both fractions HJv1 and HJv2 were pooled together as they showed similar compositions. The combined powder was further purified with PTLC, eluted with hexane/EtOAc (9.5 : 0.5), and yielded 115 mg of powder (**JF3**).

#### 2.5.3. Gas Chromatography-Mass Spectrometry (GC-MS) Analysis

The chemical composition of each of the oily fractions (JF1 and JF2) and of JF3 (obtained from the hexane extract of *J. variegata* fruit) was determined by GC-MS at Ain Shams University, Faculty of Pharmacy (Egypt). A Shimadzu GCMS-QP2010 SE system (Kyoto, Japan) operating in EI mode (70 eV) equipped with a flame ionization detector (FID), a RTx-5 (dimensions: 30 m × 0.25 mm, film thickness: 0.25 *μ*m) capillary column, and a split-splitless injector were employed for the GC-MS analysis. The initial column temperature was kept at 50°C for 3 minutes (isothermal), programmed to reach 200°C at a rate of 15°C increase every minute, and then kept constant at 200°C for 5 min (isothermal). Then, the temperature was further programmed to go up to 240°C at a rate of 3°C increase every minute and then kept constant at 240°C for 10 min (isothermal). Finally, the temperature was programmed to reach 300°C at a rate of 4°C increase per minute and then kept constant at 300°C for 10 min (isothermal). The temperature of the injector and detector was maintained at 280°C. The oven temperature was set at 45°C for 2 minutes, then was gradually increased to 300°C at a rate of 5°C/min, and then held constant at 300°C for 5 min. Diluted samples (1% v/v) were automatically injected in split mode (split ratio 1 : 15) using an autoinjector Shimadzu AOC-20i, utilizing helium as a carrier gas at a flow rate of 1.41 ml/min. All mass spectra were recorded via computerized integration. The analyses were recorded at the following conditions: filament emission current of 60 mA, ionization voltage of 70 eV, and an ion source set at 220°C.

#### 2.5.4. Identification of Compounds

Components of the oily fractions JF1 and JF2 were identified by GC based on retention time (RT) for GC, while the interpretation of mass spectra was achieved by comparing the spectral fragmentation obtained with the National Institute Standards and Technology (NIST) library database. Retention time, names of compounds, molecular weight, molecular formula, and percentage for each chemical component in the two fractions were confirmed and recorded.

#### 2.5.5. Physical and Spectral Data of Methyl Elaidate (JF3)

Methyl elaidate was obtained as a white amorphous powder (115.0 mg) that dissolves in EtOAc. The R_*f*_ value was 0.4 when using hexane/EtOAc as an eluant (9.5 : 0.5), and the melting point (m.p.) was found to be 9.5–11.5°C, which is comparable to what was found in a previous report [15]. The spectral data of the compound were as follows: GC-MS for C_19_H_36_O_2_*m/z*: 296 [M^+^, C_19_H_36_O_2_]^+^, 264 [M-OCH_4_]^+^, 222 [M-OC_4_H_10_]^+^, 180 [M-C_6_H_13_O]^+^, 137 [M-C_9_H_19_O_2_]^+^, 123 [M-C_10_H_21_O_2_]^+^, 97 [C_6_H_9_O]^+^ 87 [C_4_H_7_O_2_]+, 69 [C_3_HO_2_]^+^, 55 [base peak, C4H7]^+^, 43 [C_3_H_6_]^+^, and 41 [C_3_H_5_]^+^; ^1^H NMR (DMSO, 400 MHz): *δ* 0.86 (3H, *t*, *J* = 12 Hz, H-18), 1.26 (14H, *s*, H-17, H-16, H-15, H-14, H-13, H-4, H-5), 1.54 (4H, *t*, *J* = 10.4, 22.4, H-3, H-6), 2.04 (2H, *dd*, *J* = 5.3, 12 Hz, H-11), 2.31 (6H, *t*, *J* = 11.6 Hz, H-12, H-7, H-2), 2.76 (2H, *t*, *J* = 6.4, 16.0 Hz, H-8), 3.58 (3H, *s*, 19), and 5.33 (2H, *dd*, *J* = 8, 15 Hz, H-9, H-10); and ^13^C NMR (DMSO, 100 MHz): *δ* 14.34 (C-18), 22.41 (C-17), 24.85 (C-3, C-6), 25.66 (C-8), 27.05 (C-11), 28.87 (C-16), 29.17 (C-13, C-14), 29.39 (C-5), 31.35 (C-15), 31.72 (C-4), 33.72 (C-2, C-7, C-12), 51.61 (C-19), 128.22 (C-9), 130.18 (C-10), and 174.14 (C-1).

### 2.6. Acute Oral Toxicity of the Extract

Acute oral toxicity of the methanol extract of *J. variegata* fruit was determined in female albino rats according to previous work [16]. Briefly, five groups (six rats each) were subjected to overnight fasting and then were orally administered with the fruit extract dissolved in distilled water (with 3% Tween 80) at doses of 250, 500, 1,000, 2,000, and 3,000 mg/kg body weight (b.w.), respectively. The animals were kept under constant observation after dosing for 48 h. Signs of toxicity and/or mortalities, if any, were recorded.

### 2.7. Antifertility Study

#### 2.7.1. Abortifacient Activity

The plant extract was tested in female Albino rats for its abortifacient activity according to the method described by Khanna and Chaudhury [17] with few modifications. Females with normal estrus cycle were caged with males of proven fertility in the ratio of 2 : 1 (female: male), in the evening of the proestrous stage, and were examined in the next early morning for evidence of copulation. Rats showing a thick clump of spermatozoa in their vaginal smear in the next early morning were separated, and that day was designated as day one of pregnancy. Selected rats were randomly divided into three groups consisting of six animals each. Group I received vehicle only (3% tween-80) and served as control, and groups II and III received fruit extract at doses of 150 and 300 mg/kg, respectively, from day 11 to day 15 of pregnancy. Food was held the night before the day of experimentation. On the 10th day of pregnancy, the animals were intraperitoneally (i.p.) anesthetized using xylazine (10 mg/kg) and ketamine (60 mg/kg) under sterile conditions. The two horns of the uteri were examined to determine the implantation sites. Then, the abdominal wound was sutured in layers. Postoperational care was taken to avoid any infection. The animals were allowed to undergo full-term pregnancy. After delivery, the pups were counted and the antifertility activity of the extract was evaluated. Litters were examined for any malformation.

#### 2.7.2. Estrogenic and Antiestrogenic Activities

Estrogenic and antiestrogenic activities of the methanol extract were assessed according to Shinde et al. [18] with some modifications. Female rats were monitored for seven consecutive days for a normal estrus cycle using the vaginal smear method. The animals were anesthetized using xylazine (10 mg/kg) and ketamine (60 mg/kg) before left-side ovariectomy was performed. The left ovary was carefully dissected out from surrounding fatty tissue and dried by soaking on filter paper and weighed. The ovariectomized rats were randomly taken and divided into four experimental groups (*n* = 6). Group I was treated with vehicle (3% Tween-80) and served as control, group II was treated with estradiol valerate (0.1 mg/rat) and served as the reference group, group III was treated with the plant extract (300 mg/kg), and group IV was treated with a combination of estradiol valerate (0.1 mg/rat) and fruit extract (300 mg/kg). All groups were treated in divided doses for four consecutive days. On the eleventh day, the remaining right-sided ovaries were dissected out from all animals, properly cleaned, and dried before their respective weights were recorded. The ovaries' weight variations prior to and after treatment with *J. variegata*, estradiol valerate, and the combination were calculated. Percentage increase in ovarian weights was also calculated using the following equation.

(1) $$\begin{array}{c} \%\text{ i}\mathrm{ncrease}\;\mathrm{in}\;\mathrm{ovarian}\;\mathrm{weight}=\frac{\mathrm{weight}\;\mathrm{of}\text{ the }\mathrm{right}\;\mathrm{ovary}-\mathrm{weight}\;\mathrm{of}\text{ the }\mathrm{left}\;\mathrm{ovary}}{\mathrm{weight}\;\mathrm{of}\text{ the }\mathrm{left}\;\mathrm{ovary}}\times 100. \end{array}$$

#### 2.7.3. Histopathology

Histopathological studies were performed to investigate the effects of methanol extract of *J. variegata* fruit on ovarian histology and follicular development using the previously described technique [19]. Isolated ovaries of six animals from each group were used for histological analyses. The ovaries were fixed in 10% neutral formalin, dehydrated in graded ethanol, cleared in xylene, embedded in paraffin, and blocked out. Paraffin sections (3–6 *μ*m) were stained and examined under a light microscope. The general histological appearance of the ovary was assessed.

### 2.8. Statistical Studies

The data were analyzed using Statistical Package for Social Sciences (SPSS) version 20. The results were presented as means ± standard deviations (SEM). The data were analyzed using one-way ANOVA followed by a post hoc test; Tukey. *P* < 0.05 was considered to be statistically significant.

## 3. Results

### 3.1. GC-MS Analysis of JF1 and JF2

The GC-MS analysis of the oily fraction JF1 of the hexane extract of *J. variegata* fruit identified the presence of 27 phytoconstituents (Table 1). The most abundant components were fatty acid esters (43.77%) with the major compounds being methyl linolate (17.26%), methyl stearate (8.97%), methyl elaidate (6.35%), and methyl palmitate (4.64%). Hydrocarbon alkanes were also found and constituted 20.65% of the oil, with octacosane (5.62%), heptacosane (4.88%), and tetracosane (2.89%) being the major alkanes. Terpenoids (4.65%) were minor constituents (2.22% phytol acetate and 1.59% squalene among others).

JF2 (30 compounds, Table 2) was largely made up of fatty acids and their esters (29.47%), terpenoids (28.8%), and phytosterols (10.49%). The most abundant terpenoids were *α*-amyrin (15.48%) and cycloartenol 3-acetate (5.15%), *β*-amyrin (3.69%), and cholesta-3,5-dien-7-one (2.36%), while the major fatty acids were palmitic acid (5.48%), oleic acid (4.58%), hexanoic acid (4.55%), and methyl palmitate (4.36%). Phytosterols mainly included stigmast-4-en-3-one (7.75%) and *γ*-sitosterol (1.78%).

### 3.2. Compound (JF3): Methyl Elaidate

**Figure 1:**
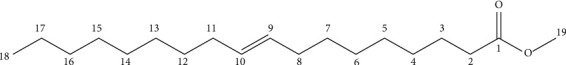
Chemical structure of methyl elaidate.

A compound (**JF3**) was isolated as a yellow oily substance, with a R_*f*_ value of 0.40 in hexane:EtOAc (9.5 : 0.5), and a melting point of 9.5–11.5°C. The molecular formula was C_19_H_36_O_2_ as indicated by a parent molecular ion [M]^+^ at *m/z* 296 in the mass spectrum obtained from GC-MS. The ^1^H-NMR spectrum in DMSO (Table 3) suggests the presence of deshielded protons of an allylic group (H-9 and H-10), which occurred at *δ*5.33 and a singlet signal of a proton bearing oxygen at *δ* 3.58 (3H, s). Sharp singlets at *δ*1.26 suggested the presence of 14 protons on 7 methylenes. A triplet peak at *δ* 2.34 with a *J* value of 11.6 Hz indicated the presence of two protons of a–CH2COO group. Another triplet signal at *δ* 0.86 with a *J* value of 12.0 Hz indicated the presence of three protons of a methyl group attached with methylene. The ^13^C-NMR assignments (shown in Table 3) based on the 2D experiments and the comparison of these data with those reported for methyl elaidate [15] confirmed that JF3 is indeed methyl elaidate (Figure 1).

### 3.3. Acute Oral Toxicity of the Extract

Observation during the period of the acute toxicity study revealed that all the examined doses (250, 500, 1,000, 2,000, and 3,000 mg/kg b.w.) of *J. variegata* fruit did not induce demonstrable acute toxic effects or deaths in all treated groups. The only changes noted were on the first day of the observational period and included drowsiness and some reduction in motor activity in animals treated with higher doses (2,000 and 3,000 mg⁄kg). All groups showed normal behavior on the second day.

### 3.4. Antifertility Activity

#### 3.4.1. Abortifacient Activity

A dose-dependent abortifacient effect was observed with the 150 and 300 mg/kg extract of *J. variegata* fruit, recording a 50% and 93% abortifacient activity, respectively (Table 4), while the fetal loss was not observed in control animals. Also, the number of litters delivered in both tested groups decreased, scoring mean number of litters of (3.33 ± 1.09) and (0.50 ± 0.34), respectively, compared to the mean number of litters recorded in the control group (5.17 ± 1.30). However, only the group treated with the 300 mg/kg extract had a significant (*P* < 0.05) reduction in number of litters born. The number of litters resorbed in both tested groups was significantly higher (*P* < 0.05) in comparison with the control group.

#### 3.4.2. Estrogenic and Antiestrogenic Activities

On day 11 postovariectomy, the results obtained in this study revealed a significant increase in the weights of the right-side ovaries of the control group (22.77%) compared to the weights of the left ovaries (Table 5). When estradiol valerate was administered (at a dose of 0.1 mg/rat/day), that resulted in a significant increase (*P* < 0.01) in ovarian weight as compared to that of the control group. Interestingly, the 300 mg/kg administered test group also produced a significant (*P* < 0.01) elevation in the percentage increase in ovarian weight by 66.86% compared to the weight in the control group. In addition, a combination dosing of 300 mg/kg *J. variegata* and 0.1 mg/rat/day estradiol valerate resulted in a significant (*P* < 0.05) increase in ovarian weight (53.80%) but the increase in weight was statistically insignificant when compared with the ovarian weight increase seen in the estradiol valerate group. All groups showed a reduction in body weight after the treatment period compared to their initial body weights recorded before treatment (Table 5).

#### 3.4.3. Histopathological Studies

**Figure 2:**
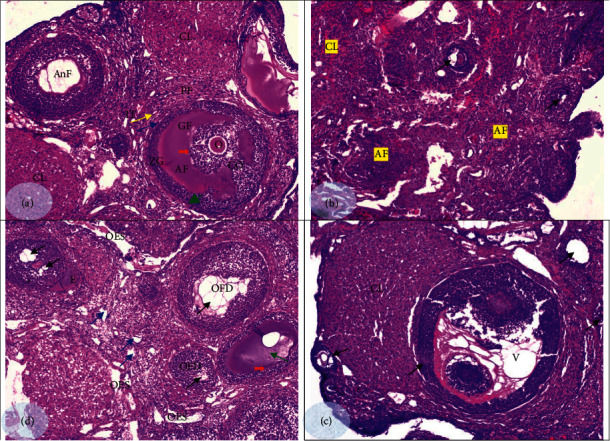
Histopathological studies on rat ovaries. (a) Ovary from negative control group: primary follicle (PF), antral follicles (AnF), Graafian follicle (GF), oocyte (O), corona radiata (red arrow), zona granulose (ZG), basement membrane (green arrow), theca externa (yellow arrow), theca interna (blue arrow), cumulus oophorous (CO), and interstitial tissue (IT). (b) Ovary from rats treated with estradiol valerate: vacuolated oocytes (arrow), atretic follicles (AF), and corpus luteum (CL). (c) Ovary from rats treated with plant extract (300 mg/kg): shrinkage and shift of ooplasm to one side (green arrow), absence of nucleus and zona pellucida (red arrow), cavities surrounding the ovum (black arrows), vacant areas in the ovary stroma (blue arrows), only one follicle showed nucleus (∗), OES: optical empty spaces, OFD: ovarian follicle denudation, and E: edema. (d) Ovary from rats treated with plant extract plus estradiol: degenerative different ovarian follicles (arrows) and disruption of the area that surrounded the ovum (∗) and vacuolated the antrum (V) (H&E, ×100).

The histopathological study of methanol extract of *J. variegata* fruit was carried out to evaluate the extract effect on the number and the development of the right-sided ovarian follicles (Figure 2). The histological analysis of the control group ovaries showed multiple normal healthy ovarian follicles. There were five follicles at different stages of development; a primordial follicle, a primary follicle; an antral follicle, a secondary follicle, and a fully developed Graafian follicle (Figure 2(a)). In addition, control rats' ovaries contained a mature Graafian follicle with an oocyte inside surrounded by the follicular cells the corona radiata. The zone of granulosa cells was well defined, and the basement membrane was distinguished. Each follicle was surrounded by both theca externa and theca interna cells. The ovum was attached to membrana granulosa by cumulus oophorous. All seen follicles were found within the interstitial tissue of the ovary. There was no degenerating follicle, hemorrhagic follicle, degenerating corpora lutea, or any other abnormal features. However, estradiol valerate-treated animals had a significantly lower number of primordial follicles, primary follicles, secondary follicles, and Graafian follicles compared to the control group. Also, multiple degenerated ovarian follicles with vacuolated oocytes were observed. There was also an increase in the number of atretic follicles and a decrease in the number of corpora lutea within ovaries (Figure 2(b)). Ovarian follicular development upon treatment with the plant extract is shown in Figure 2(c).

Ovarian follicles may degenerate when their normal growth, and differentiation processes are disrupted. In this experiment, administering a 300 mg/kg extract to rats resulted in the generation of degenerated underdeveloped ovarian follicles at different stages (primordial, primary, secondary, and Graafian stages) compared to the follicles produced in normal control rats. A significant increase in the number of atretic follicles was also noted in treated rats and not a single follicle contained a healthy intact ovum or a normal nucleus. There were also clear degenerative signs in the ovum including nonvisibility of nucleus, shrinkage, and shift of ooplasm to one side and the absence of zona pellucida. The area surrounding the ovum showed multiple cavities. In the stroma of the ovary, many vacant areas were also observed. Indeed, the typical overall appearance of a follicle has been totally lost. In all the four sections taken, all the examined follicles were devoid of nuclei except one, and several optical empty spaces, ovarian follicle denudation, and edema were also observed. Similarly, the ovaries of rats treated with both the plant extract and estradiol valerate had a lower number of developing ovarian follicles that were characterized by clear degenerative changes (Figure 2(d)). Finally, there was a decrease in the number of corpora lutea in the ovary, and each corpus was characterized by a disrupted histological appearance and had a clearly vacuolated antrum.

## 4. Discussion

Several herbs traditionally used in certain rural areas around the globe for fertility regulation have been shown to contain phytoconstituents such as steroids, flavonoids, terpenoids, alkaloids, and steroidal saponins, which may be responsible for the observed antifertility activity [20]. However, no studies have been reported addressing the antifertility activity of *J. variegata*. This study reveals that *J. variegata* contained oils with various bioactive constituents that may play a role in the antifertility activity exerted by the plant. The nature and composition of the oils found in *J. variegata* fruit are described for the first time in this study as they have not been reported elsewhere. The chemicals found in the oils were fatty acids and fatty acid esters, hydrocarbon alkanes, terpenoids, and phytosterols. The presence of fatty acids and their esters is in accordance with those reported for other *Jatropha* species such as *J. curcas* and *J. glauca* [21]. Methyl elaidate, an unsaturated fatty acid, has been isolated from some medicinal plants but not from *Jatropha* [15, 22]. Methyl elaidate is regarded as an apoptosis inducer in cancer cells [23]. Also, phytosterols play major roles in pharmaceuticals like the production of therapeutic steroids, in nutrition (as cholesterol-lowering and antineoplastic additives in certain food products), and in cosmetics [24]. In addition, *α*-amyrin, a major compound found in this study in the oil of *J. variegata*, maybe for the source of the shown antifertility effect of this plant as reported previously by another group in male albino rats [25].

Notably, the *J. variegata* fruit extract did not induce toxicological effects nor did it result in behavioral changes in rats, even at the highest dose administered (3,000 mg/kg). This indicates that even a dose as high as 3,000 mg/kg of the extract is considered nontoxic in rats because no adverse effects were observed during the observational period [26]. Therefore, one-tenth [27] of that dose or less, i.e., 150 mg/kg and 300 mg/kg body weight, was chosen to carry out the subsequent antifertility testing studies.

Confirming its abortifacient activity, both doses (150 and 300 mg/kg) of *J. variegata* extract resulted in a dose-dependent increase in the number of lost implants and in the number of resorbed fetuses in female rats. The plant extract also exhibited a significant abortifacient activity, particularly at a dose of 300 mg/kg. A higher number of lost implants and a higher resorption index are considered indices of decreased fertility and predict the failure of a female to carry a viable embryo [28]. Such high occurrence of fetal resorptions in a female genital tract suggests that the interruption of pregnancy occurred following successful embryo implantation. In accordance with these findings, a previous investigation on another species of *Jatropha* (*J. gossypifolia*) revealed that the extract of the plant also exerted a significant abortifacient activity [29].

The potential abortifacient effect of the extract of *J. variegata* fruit might be mediated through induction of an imbalance in estrogenic vs. progestogenic activities, since a finely regulated equilibrium of these hormones is required for maintenance of pregnancy [30]. Thus, phytoconstituents present in a plant that could disrupt the estrogen to progestin ratio in a female may hinder the development of the embryo and eventually induce miscarriage [31]. This study revealed that the *J. variegata* fruit extract possessed a significant estrogenic activity. As abortion in females is usually a result of estrogen/progestin level abnormalities [28], it is thus possible that the abortifacient activity of the plant is induced by its reservoir of phytochemicals with estrogenic activity. Indeed, compounds with known estrogenic and/or progestogenic activity could disturb the hormonal milieu in the uterus, disturb fetal implantation and growth, and may induce miscarriage [32].

In the estrogenic/antiestrogenic study, administration of the *J. variegata* fruit extract led to an increase in ovary weight in mature female rats, indicating an estrogenic activity of the extract. The change in weight was significant compared to the negative control and similar to that caused by the standard group that received estradiol valerate. This indicates that *J. variegata* fruit possesses an estrogenic effect that simulates the effect produced by estradiol valerate, a well-known and widely used estrogen. However, when the fruit extract was given in combination with estradiol valerate, a slight antiestrogenic activity was observed. This indicates that the extract may have acted as a partial estrogen receptor agonist and thus took the role of a competitive antagonist in the presence of the much more potent estradiol valerate [33, 34].

The loss in body weight after the dosing period in all tested and control groups may be due to a prodiarrheal side effect observed during the dosing period, which was a nonanticipated unwelcomed issue encountered in this study. Indeed, this side effect was also found in *J. curcas* fruit during a previous investigation of its fertility regulatory activity [35].

During each menstrual cycle, two phases of elevated estrogen levels can be distinguished: once during the proliferative follicular phase, where the growing ovarian follicles secrete increasing amounts of estradiol (E2) that peak 1 day before ovulation, and another one during the luteal phase; after ovulation, the corpus luteum secretes significant amounts of estrogens, in addition to progesterone [36, 37]. Plasma estradiol concentrations elevated during the proliferative phase activate positive estradiol feedback at the level of both the pituitary and hypothalamus resulting in a mid-cycle luteinizing hormone (LH) surge, which is essential for ovulation [38, 39]. Therefore, any changes that significantly affect these hormonal cues result in anovulation [38]. This means that estrogen plays a significant role in controlling the ovulatory cycle in female mammal [40]. In addition to the roles of estradiol and LH in menstrual cycle regulation, follicle-stimulating hormone (FSH) also plays an important role in promoting the development of the immature ovarian follicles, which leads to the increase in estrogen production from the ovary, and this estrogen in turn causes the subsequent positive feedback effect that leads to the LH surge and ovulation mid-cycle [38, 39]. On the other hand, a feedback inhibition of GnRH secretion may be induced by excess exogenous estrogen that influences the production of FSH from the pituitary gland. So, if FSH is sufficiently suppressed by estrogen, follicular growth will be suppressed [39, 40]. If Graafian follicles do not grow and mature, ovulation would not occur, nor would conception and pregnancy [40], and this is exactly the basis for the majority of widely used estrogen-containing hormonal contraceptives [39, 41].

Thus, it is possible that *J. variegata* fruit estrogenic activity works in a similar fashion, that is, via induction of a negative feedback that results in hypothalamic suppression, and, hence, inhibits the secretion of FSH and LH, and eventually failure to ovulate. This may also explain the anticonceptive effect of the plant observed in this study. Plants possessing estrogenic effects have been reported to possess significant antifertility activity [20], and *J. variegata* fruit likely similarly acts. In line with our findings, a previous investigation on other species of *Jatropha* like *J. gossypifolia* has reported that *J. gossypifolia* extracts possessed significant estrogenic activity [29]. In addition to the antiovulatory effect, the estrogenic activity of *J. variegata* in this study may also explain, at least in part, the postcoital anti-implantation and abortifacient activity observed by locals in Yemen. Estrogenic substances are known to increase uterine contraction, which in turn could expel a fertilized egg from the uterus and thus cause abortion [30]. Furthermore, as in the implantation of embryos in uterus walls, maintenance of pregnancy requires a delicate equilibrium of estrogen and progesterone and any disturbance in the levels of these hormones may induce abortion [20, 30]. Taken together, this study has revealed that the extract of *J. variegata* fruit possesses estrogenic activity that justifies the folkloric claim of the plant fruit acting as an oral contraceptive.

Fertilization in a female depends on the availability and maturation of the ovarian oocyte [42]. Atretic follicles are degenerating follicles, where ovarian follicles degenerate as a result of disruption of their growth and differentiation leading to infertility [43, 44]. In this study, histopathological analyses of right ovaries at day 11 post-treatment revealed marked changes in follicular growth and histology in the extract-treated rats. Although ovaries in the control group showed the various types of follicles at all stages of folliculogenesis (primordial, primary, secondary, and mature follicles), the number of primordial, primary, secondary, and Graafian follicles was significantly lower in the extract-treated groups and in the estradiol valerate-treated group. In addition, degeneration signs were clearly observed in several atretic follicles seen in the test groups. This suggests compromised folliculogenesis caused by the *J. variegata* extract and supports the estrogenic activity of the plant.

The increasing number of atretic follicles showed in our findings indicates that there were certain phytocompounds in the plant extract, which affected normal folliculogenesis [45]. These compounds must have a definite estrogenic effect that altered the steroidal sex hormone levels. As known, an optimal blood level of FSH is a prerequisite for the initiation and maintenance of normal ovarian folliculogenesis. Therefore, the ovarian follicle atresia observed in this study may have been induced by the estrogen-caused reduction in serum FSH levels. The histological findings of this study suggest a hypothalamic-pituitary-gonadal axis dysfunction after treatment with the plant extract through a negative feedback mechanism of the gonadotropins FSH and LH as previously explained [43]. In accordance, several plants have been shown to act in a similar fashion [43, 46–52].

Changes in oocyte growth and maturation are influenced by gonadotropins, sex hormones, and growth factors [45]. As reported in this study, the *J. variegata* extract caused the rats' ova to show clear degenerative changes; the unnucleated ovum showed a shrinkage in size, and the ooplasm was shifted to one side, possibly due to induced apoptosis of granulosa cells caused by the extract. Also, the ova were detached from the periphery possibly due to fragmentation of the inner granulosa cells. These events were followed by thinning of cumulus oophorous, freeing of the oocyte into the liquor, and finally sloughing of the ovum and entire granulosa, which gave the appearance of vacant areas that were largely observed in the stroma of the ovary [51]. Therefore, a disruption of the structural complementarity of oocyte and surrounding cellular cells is responsible for compromised folliculogenesis and oogenesis, which eventually may lead to infertility [53].

The zona pellucida (ZP) that surrounds the plasma membrane of the oocyte is a relatively thick extracellular coat made up of glycoproteins. It is synthesized and secreted by the growing oocyte itself and laid down during the final stages of oogenesis when growing oocytes enter their growth phase [53, 54]. The absence of zona pellucida is another change seen in this study. Rats with fully grown oocytes and ovulated eggs but lacking a zona pellucida lose fertility [53]. It is plausible that there is damage to the ovum or shrinkage in size, and the absence of zona pellucida and massive apoptosis in granulosa cells are all associated with infertility [51]. In this study, it can be inferred that the disintegration of ova in the ovaries is a specific effect of *J. variegate* fruit.

## 5. Conclusions

From this study, it is concluded that the methanol extract of *J. variegata* fruit possesses a significant antifertility activity via exerting a potent estrogenic effect and an abortifacient activity in a dose-dependent manner. This activity could be attributed to certain estrogenic phytoconstituents naturally present in the plant. These encouraging findings described in this study provide an insight into the traditional usage of *J. variegata* as a contraceptive for women in Yemen and may potentially be exploited in man's pursuit for safer natural contraceptives.

## Figures and Tables

**Table 1:** Phytoconstituents in the oily fraction (**JF1**) of the hexane extract of *J. variegata* fruit identified by GC-MS.

No.	RT	Name of the compound	MF	MW	% Peak area
1	12.37	Pentadecane	C_15_H_32_	212	0.78
2	15.27	1-Nonadecene	C_19_H_38_	266	0.65
3	17.35	Methyl palmitate	C_17_H_34_O_2_	270	4.64
4	21.13	Methyl linolate	C_19_H_34_O_2_	294	17.26
5	21.24	Methyl elaidate	C_19_H_36_O_2_	296	6.35
6	21.84	Methyl stearate	C_19_H_38_O_2_	298	8.97
7	22.84	Ethyl elaidate	C_20_H_38_O_2_	310	0.56
8	26.12	Heneicosane	C_21_H_44_	296	0.93
9	26.944	Methyl arachidate	C_21_H_42_O_2_	326	0.81
10	28.724	Eicosane	C_20_H_42_	282	1.56
11	31.33	Octacosane	C_28_H_58_	394	5.62
12	32.194	Methyl docosanoate	C_23_H_46_O_2_	354	0.99
13	32.80	Z-6,17-Octadecadien-1-ol acetate	C_20_H_36_O_2_	308	0.5
14	34.17	Tetracosane	C_24_H_50_	338	2.89
15	35.283	Methyl tricosanoate	C_24_H_48_O_2_	368	0.59
16	37.75	Heptacosane	C_27_H_56_	380	4.88
17	39.189	Methyl tetracosanoate	C_25_H_50_O_2_	382	1.4
18	39.96	Dioctyl isophthalate	C_24_H_38_O_4_	390	3.12
19	42.202	Tetratriacontane	C_34_H_70_	478	1.36
20	43.481	Squalene	C_30_H_50_	410	1.59
21	43.7	Methyl pentacosanoate	C_26_H_52_O_2_	396	0.56
22	46.048	Tetratetracontane	C_44_H_90_	618	1.3
23	47.239	Methyl hexacosanoate	C_27_H_54_O_2_	410	0.68
24	49.098	Tetratetracontane	C_44_H_90_	618	0.68
25	50.814	*α*-Amyrin	C_30_H_50_O	426	0.84
26	61.307	Nonadecatriene-5,14-diol	C_19_H_34_O_2_	294	0.96
27	61.534	Phytol acetate	C_22_H_42_O_2_	338	2.22

**Table 2:** Phytoconstituents in the oily fraction (**JF2**) of the hexane extract of *J. variegata* fruit identified by GC-MS.

No.	RT	Name of the compound	MF	MW	% peak area
1	6.8	2-Amylfuran	C_9_H_14_O	138	0.58
2	7.084	Hexanoic acid	C_6_H_12_O_2_	116	4.55
3	9.226	Octanoic acid	C_8_H_16_O_2_	144	1.1
4	10.208	Nonanoic acid	C_9_H_18_O_2_	158	0.63
5	11.034	8-Methyl-1-undecene	C_12_H_24_	168	0.92
6	11.071	6-Methyl octadecane	C_19_H_40_	268	1.18
7	11.178	*N*-Decanoic acid	C_10_H_20_O_2_	172	0.6
8	11.846	Methyl 9-oxononanoate	C_10_H_18_O_3_	186	0.59
9	12.423	Oleic acid	C_18_H_34_O_2_	282	4.85
10	13.25	Azelaic acid	C_9_H_16_O_4_	188	0.74
11	13.99	2-*n*-Octylfuran	C_12_H_20_O	180	1.53
12	14.97	Tetradecanoic acid	C_14_H_28_O_2_	228	0.56
13	17.35	Methyl palmitate	C_17_H_34_O_2_	270	4.36
14	18.29	Palmitic acid	C_16_H_32_O_2_	256	5.48
15	21.21	(E)-Methyl octadec-9-enoate	C_19_H_36_O_2_	296	2.79
16	21.80	Methyl stearate	C_19_H_38_O_2_	298	2.98
17	22.39	cis-Vaccenic acid	C_18_H_34_O_2_	282	0.64
18	24.55	(E)-13-Docosenoic acid	C_22_H_42_O_2_	338	0.93
19	32.83	Bis(2-ethylhexyl) phthalate	C_24_H_38_O_4_	390	0.93
20	39.90	Dioctyl isophthalate	C_24_H_38_O_4_	390	3.49
21	49.21	Unknown		385	1.59
22	49.63	*β*-Amyrin	C_30_H_50_O	426	3.69
23	50.87	*α*-Amyrin	C_30_H_50_O	426	15.48
24	53.29	*β*-Amyrin	C_30_H_48_O	424	0.53
25	56.39	*γ*-Sitosterol	C_29_H_50_O	414	1.78
26	56.62	Cycloartenol 3-acetate	C_32_H_52_O_2_	468	5.15
27	57.29	Cholest-4-en-3-one	C_27_H_44_O	384	0.87
28	57.99	4,22-Stigmastadiene-3-one	C_29_H_46_O	410	0.96
29	58.40	Cholesta-3,5-dien-7-one	C_27_H_42_O	382	2.36
30	59.37	Stigmast-4-en-3-one	C_29_H_48_O	412	7.75

**Table 3:** ^1^H and ^13^C NMR spectral data for methyl elaidate (JF3) in DMSO^a^.

Position	*δ*H multiplicity (*J* = Hz)	*δ*H multiplicity (*J* = Hz)∗	Cosy	HMBC	*δ*C	*δ*C∗
1			—	—	174.14	172.8
2	2.31 *t* (11.6)	2.18 m	H3	C1, C4	33.72	32.4
3	1.54 *t* (10.4, 22.4)	1.59 *t*	H2	C1, C2	24.85	24.3
4	1.26 s	1.27 s		C5	31.72	28.2
5	1.26 s	1.27 s	H6	C4	29.39	28.5
6	1.54 *t* (10.4, 22.4)	1.27 s	H7/H5	C7, C5	24.85	28.9
7	2.31 *t* (11.6)	1.30 s	H6	C8, C6	33.72	29.4
8	2.76 *t* (6.4, 16.0)	1.91 m	H9	C9, C10	25.66	32.4
9	5.33 *dd* (8, 15)	5.29 *dd*	H8		128.22	128.9
10	5.33 *dd* (8, 15)	5.29 *dd*	H11		130.18	128.9
11	2.04 *dd* (5.3, 12)	1.91 m	H10	C12, C13, C10, C11	27.05	32.4
12	2.31 *t* (11.6)	1.30 s		C13, C11	33.72	29.4
13	1.26 s	1.27 s		C12	29.17	28.9
14	1.26 s	1.27 s		C15	29.17	28.9
15	1.26 s	1.27 s		C16, C14	31.35	28.5
16	1.26 s	1.27 s		C15	28.87	30.6
17	1.26 s	1.30 s	H18		22.41	21.8
18	0.86 *t* (12.0)	0.95 *t*	H17	C17, C16	14.34	13.8
19	3.58 s	3.59 *d*		C1	51.61	49.2

a = chemical shift values are given in ppm. COSY: correlation spectroscopy; HMBC: heteronuclear multiple bond correlation; ∗compared to [15].

**Table 4:** Effect of the methanol extract of *J. variegata* fruit on litter size and resorption index of female rats.

Treatment	Implants	Litters	Resorption	% abortifacient activity
Control	5.17 ± 1.30	5.17 ± 1.30	0.00 ± 0.00	00.00
*J. variegata* (150 mg/kg)	6.67 ± 1.20	3.33 ± 1.09	3.33 ± 0.67∗∗	50.00
*J. variegata* (300 mg/kg)	7.17 ± 0.833	0.50 ± 0.34∗	6.67 ± 0.99∗∗∗	93.02

*n* = 6 in each group. ^*∗*^*P* < 0.05, ^*∗∗*^*P* < 0.01, and ^*∗∗∗*^*P* < 0.001 compared to the control group. Values are expressed as means ± SEM and analyzed by one-way analysis of variance followed by Tukey's post hoc test using SPSS software.

**Table 5:** Antiestrogenic effect of the methanol extract of *J. variegata* fruit on body weight and % increase of ovarian weight in female rats.

Treatment group	Initial body weight (g)	Final body weight (g)	Weight of the ovary (mg/100 g body weight)		% increase in ovarian weight
			Left side	Right side	
Control Tween 80 (3% v/v)	223.83 ± 6.89	195.50 ± 3.85	13.44 ± 1.09	16.50 ± 1.51	22.77 ± 6.68
Estradiol valerate (0.1 mg)	202.00 ± 2.44	197.00 ± 3.51	10.75 ± 0.83	18.12 ± 0.94	71.17 ± 9.38^∗∗^
*J. variegata* (300 mg/kg)	195.80 ± 17.43	187.60 ± 9.37	13.45 ± 0.45	22.42 ± 1.22	66.86 ± 8.53^∗∗^
*J. variegata* (300 mg/kg) + estradiol valerate	200.83 ± 1.73	196.50 ± 10.15	11.18 ± 1.45	17.89 ± 1.32	53.80 ± 8.76^∗^

*n* = 6 in each group. Values are expressed as means ± SEM. ^*∗*^*P* < 0.05 and ^*∗∗*^*P* < 0.01 compared to the control group. Data were analyzed by one-way analysis of variance followed by Tukey's post hoc test using SPSS software.

## Data Availability

The data used to support the findings of this study are included within the article.

## Supplementary Materials

Supplementary Materials The ARRIVE guidelines 2.0: author checklist.
